# Transcriptome Sequencing of Lima Bean (*Phaseolus lunatus*) to Identify Putative Positive Selection in *Phaseolus* and Legumes

**DOI:** 10.3390/ijms160715172

**Published:** 2015-07-03

**Authors:** Fengqi Li, Depan Cao, Yang Liu, Ting Yang, Guirong Wang

**Affiliations:** State Key Laboratory for Biology of Plant Diseases and Insect Pests, Institute of Plant Protection, Chinese Academy of Agricultural Sciences, Beijing100193, China; E-Mails: pandit@163.com (F.L.); depancao@163.com (D.C.); yangliu@ippcaas.cn (Y.L.); tyang@ippcaas.cn (T.Y.)

**Keywords:** legume, lima bean, transcriptome, positive selection

## Abstract

The identification of genes under positive selection is a central goal of evolutionary biology. Many legume species, including *Phaseolus vulgaris* (common bean) and *Phaseolus lunatus* (lima bean), have important ecological and economic value. In this study, we sequenced and assembled the transcriptome of one *Phaseolus* species, lima bean. A comparison with the genomes of six other legume species, including the common bean, *Medicago*, *lotus*, soybean, chickpea, and pigeonpea, revealed 15 and 4 orthologous groups with signatures of positive selection among the two *Phaseolus* species and among the seven legume species, respectively. Characterization of these positively selected genes using Non redundant (nr) annotation, gene ontology (GO) classification, GO term enrichment and Kyoto Encyclopedia of Genes and Genomes (KEGG) pathway analyses revealed that these genes are mostly involved in thylakoids, photosynthesis and metabolism. This study identified genes that may be related to the divergence of the *Phaseolus* and legume species. These detected genes are particularly good candidates for subsequent functional studies.

## 1. Introduction

The discovery of genes that are influenced by natural selection is a central goal of evolutionary biology. One widely used method for inferring the targets of positive Darwinian selection is to test the ratio, numbers, and patterns of nonsynonymous and synonymous substitutions in homologous protein coding regions. This approach has been widely employed to infer the selection of single genes and functional classes of genes, including on a genome-wide scale [1,2,3,4,5,6,7].

The substitution ratio (*k*_a_/*k*_s_ value, ω) between non-synonymous (*k*_a_) and synonymous (*k*_s_) substitutions per site is often used to identify evidence for adaptive evolution [8,9]. Generally, ω > 1 is interpreted as an indicator for positive selection, ω < 1 indicates purifying selection, and ω = 1 suggests neutral evolution.

Understanding the adaptive evolution of species has long been limited by the lack of genome sequences. In recent years, advances in next-generation sequencing have made it possible to identify positively selected genes using more extensively sampled species. For example, Backstrom *et al.* [6] identified genes of house finch undergoing positive selection in passerine birds using house finch transcriptome data. Zhang *et al.* [3] compared orthologous groups (OGs) between two bamboo species and 14 grass species and identified genes that showed putatively positive selection by combined analysis of transcriptomic and genomic data. They found that the positively selected genes were distributed among several functional classes and include genes involved in biotic and abiotic stress response, reproduction and development and plant metabolism [3]. Moreover, Xia *et al.* [10] detected 211 positively selected OGs between the tea tree species *C. oleifera* and *C. sinensis* by comparative transcriptomics. These OGs were found to be mainly involved in oxidation-reduction processes, integral to the membrane and ATP binding [10]. Because few studies have conducted positive selection analysis using plant genomic datasets [3,11,12], this type of knowledge is still limited in legumes, particularly in *Phaseolus*.

Legumes play a critical role in maintaining ecosystem nitrogen cycling. Several legume species are precious resources for the production of fibre, bioenergy, medicine and chemicals. Legumes are also regarded as a model for plant biology study [13]. Although they have important ecological and economic value, the evidence of positive selection among the legumes is still not well understood. The genomes of six legume species, barrel medic (*Medicago truncatula*), birds foot trefoil (*Lotus japonicus*), soybean (*Glycine max*), chickpea (*Cicer arietinum*), pigeonpea (*Cajanus cajan*) and common bean (*Phaseolus vulgaris*), have been sequenced [14,15,16,17,18,19]. These sequencing projects provide us with an opportunity to look for evidence of positive selection in *Phaseolus* and legumes.

Originating in Central and South America, lima bean is the second most important food legume of *Phaseolus* after the common bean [20]. Lima bean has been cultivated in the Neotropics for at least 6000 years [21], and it is now cultivated in all tropical regions of the world with high genetic diversity and yield potential [22]. Although both common bean and lima bean originated in Neotropical areas, they have evolved in different ecological zones. The common bean has evolved at higher altitudes than the lima bean. It is likely that natural selection under different ecological environments has driven the evolution of both common bean and lima bean and their divergence. However, the genetic basis of their divergence and their environment adaptation remains unknown.

The genome sequence of lima bean is not yet available. In this study, we sequenced and assembled the transcriptome of lima bean. To identify genes possibly related to the divergence and environmental adaptation of *Phaseolus*, we compared OGs between lima bean and common bean and identified genes subject to positive selection. Following this, we took a broader approach and compared OGs among seven legume species, *Medicago*, *lotus*, soybean, chickpea, pigeonpea, common bean and lima bean, and identified positively selected genes in legumes. The genes identified in this study as most likely being under positive selection are good candidates for subsequent functional studies.

## 2. Results

### 2.1. Transcriptome Sequencing and De Novo Assembly of the Lima Bean Transcriptome

Four cDNA libraries were prepared and sequenced from samples of lima bean. After filtering out low-quality and adapter sequences from 24.45 Gb of raw sequence data, there were approximately 248.53 million clean reads remaining for transcriptomic analysis with 98.84% Q20 bases (Table S1). These clean reads were assembled *de novo* by the Trinity method [23], which led to 96,248 unigenes spanning a total of 87.12 Mb of sequence. The mean length and N50 of the final all-unigene set were 949 and 1795 bp, respectively. An overview of the assembly results is shown in Table 1.

**Table 1:** Statistics of unigenes of lima bean transcripts obtained from assembly

Assembly	Statistics
Number of unigenes	96,248
Large unigenes (≥1000 bp)	29,556
Max unigene length (bp)	20,020
Mean unigene length (bp)	949
N50 length (bp)	1795
Total bases (MB)	87.12

**Figure 1:**
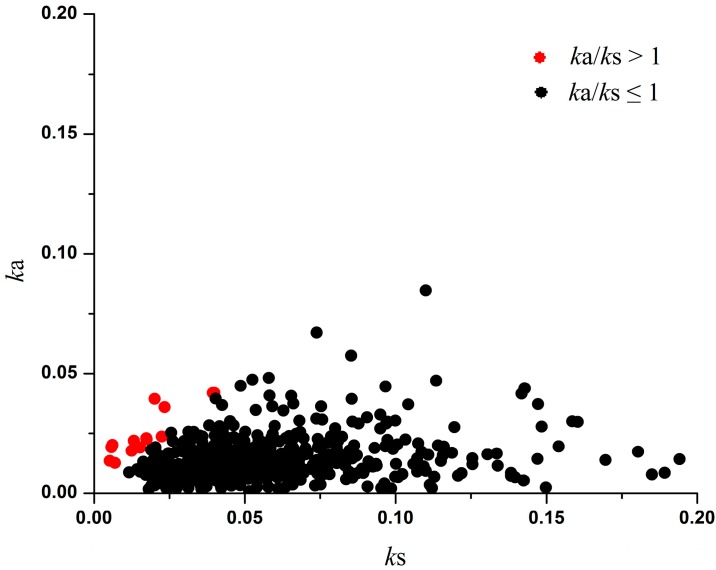
Distribution of *k*_a_ and *k*_s_ in 550 *P. vulgaris*–*P. lunatus* OGs. The threshold of *k*_a_/*k*_s_ = 1 indicates positive selection. The analysis was performed using the Yang and Nielsen method [24].

**Table 2:** Site Models for positively selected genes in seven legume species.

OGs_ID	M1a *vs.* M2a		Sites ^a^	M7 *vs*. M8		Sites ^a^	Protein Function
	LRT	*p* (fdr)		LRT	*p* (fdr)		
OG_9805	9.03 × 10^−8^	1.72 × 10^−6^	62N 89I 96L 128F 138Q	6.00 × 10^−12^	1.49 × 10^−10^	62N 89I 96L 128F 138Q	Alanine-tRNA ligase
OG_9947	4.66 × 10^−4^	0.004	320V 483Q	1.62 × 10^−4^	0.0013	320V 480S 483Q	Ceramide synthase lag1
OG_10941	0.005	0.021	21G	0.001	0.004	21G	Acyl-CoA *N*-acyltransferase-like protein
OG_10618	0.011	0.035	75W 106F	0.003	0.0074	75W 106F	Pollen Ole e 1 allergen and extension family protein
OG_9713	0.006	0.021	NS ^b^	2.28 × 10^−4^	0.0014	92I 177V	Mitochondrial inner membrane translocase, subunit tim44-related protein
OG_10037	0.003	0.019	NS	7.34 × 10^−4^	0.0028	17P 127M	Bromodomain transcription factor
OG_10186	0.022	0.058	379 A	8.80 × 10^−5^	0.0011	310V 348K 379A 516S	2,3-Bisphosphoglycerate-independent, phosphoglycerate mutase
OG_10467	0.024	0.058	32 G	0.01	0.016	32G	Uncharacterized protein
OG_10095	0.212	0.287	NS	0.021	0.0298	100S	Hnh endonuclease
OG_10235	0.030	0.062	NS	6.88 × 10^−4^	0.0028	243D 291H	Golgin candidate 2
OG_10350	0.054	0.085	NS	0.009	0.0155	180F	Pentatricopeptide repeat-containing protein
OG_10623	0.270	0.342	NS	0.006	0.0112	98P	Dessication-induced 1voc-like protein
OG_10631	1.000	1.000	NS	0.013	0.0191	323A	Endoribonuclease dicer-like 2
OG_10744	1.000	1.000	NS	0.004	0.008	489L	Transmembrane amino acid transporter family protein
OG_9360	1.000	1.000	NS	0.005	0.0106	772N	Pseudouridine synthase family protein
OG_9471	0.033	0.062	NS	0.002	0.004	28R	DnaJ domain-containing protein
OG_9474	0.432	0.513	NS	0.026	0.0352	309I 327I	Cation calcium exchanger 5
OG_9589	0.042	0.073	NS	8.14 × 10^−4^	0.0028	797D	Histone-lysine *N*-methyltransferase, H3 lysine-9 specific SUVH4
OG_9889	0.100	0.147	NS	0.039	0.0492	53M	Uncharacterized protein

^a^ When *p* values of LRT and FDR were below 0.05, positively selected sites were estimated by BEB (BEB value > 95%).; ^b^ NS, not significant.

### 2.2. Orthologous Groups among Legume Species and Evolutionary Analysis

Using OrthoMCL5 and HaMStR v13.2.3 with relatively strict filters (*-representative*, *-strict*, *-eval_limit = 0.00001*, *-rbh*), we identified 550 OGs between *Phaseolus* species (lima bean and common bean) and 413 OGs among the seven legume species.

We performed maximum likelihood analyses of *k*_a_ and *k*_s_ to identify genes that evolved under positive selection in the 550 OGs from the two *Phaseolus* species. Of these genes, 15 OGs have *k*_a_/*k*_s_ > 1 and 535 OGs have *k*_a_/*k*_s_ < 1 (Figure 1 and Table S2). According to previous reports [10,24], *k*_a_/*k*_s_ above 1 indicates a sign of positive selection, and *k*_a_/*k*_s_ below 1 suggests negative selection.

We also used site models to identify positively selected amino acid sites in the protein coding sequences based on the 413 OGs across seven species in the legume family. In total, we identified four OGs with sites under positive selection by likelihood-ratio tests (LRT), false discovery rate (FDR) and Bayes empirical Bayes (BEB) analyses in both models M1a–M2a and M7–M8 (Table 2 and Table S3). In addition, 13 OGs were subjected to positive selection in the LRT, FDR and BEB analyses of models M7–M8.

**Figure 2:**
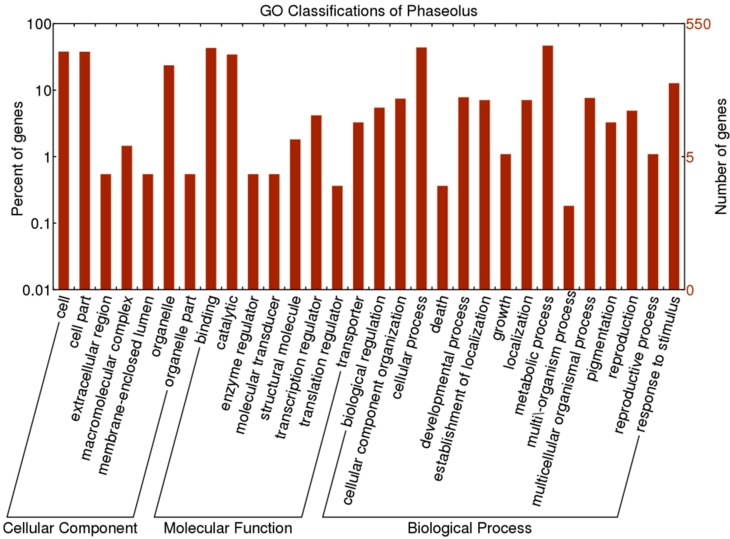
GO classifications for 550 OGs of *P. vulgaris*–*P. lunatus*

**Table 3:** The Over-Represented GO terms enriched in the test dataset compared with the reference dataset.

Gene Class	GO ID	GO Term	FWER	*p* (Fisher’s Exact Test)	Frequency ^a^ in Test Set	Frequency in Reference Set
Positive OGs between *Phaseolus*	GO:0009579	Thylakoid	0.012326	0.001462	3/15	11/422
Positive OGs between *Phaseolus*	GO:0015979	Photosynthesis	0.174733	0.018151	2/15	10/422
Positive OGs between *Phaseolus*	GO:0006091	Generation of precursor metabolites and energy	0.272852	0.0280331	2/15	13/422
Positive OGs among legumes	GO:0016746	Transferring acyl groups	0.0350915	0.00329338	2/4	8/400

^a^ The number of times the term was found/the number of OGs that were in the set.

### 2.3. Functional Categories

Through the use of non redundant (nr) annotation, gene ontology (GO) classification, GO term enrichment and Kyoto Encyclopedia of Genes and Genomes (KEGG) pathway analysis, we investigated the functional classifications for all OGs. Within 550 one-to-one OGs between the two *Phaseolus* species, protein function for each ortholog was annotated using BLASTX against the NCBI-nr protein database (Table S2). Of the 15 OGs with positive selection (ω > 1), six were related to stress response and photosynthesis, including “cold-regulated 413-plasma membrane 2”, “Seven transmembrane MLO family protein”, “Thioredoxin superfamily protein”, “photosystem I light harvesting complex gene 5 (Lhc5)”, “NAD(P)H dehydrogenase 18 (NDH18)”, and “PsbQ-like 2 (PQL2)”.

For GO annotation of the 550 one-to-one OGs, there were 422 OGs classified into 32 GO terms (Figure 2 and Table S2). Among the 15 positively selected OGs, seven were mainly involved in “cell part” within cellular component category, seven were mainly related to “cellular process” and “metabolic process” within the biological process category, and six were mainly involved in “binding” within the biological process category (Table S2).

Using the 550 OGs as the reference dataset, we detected three GO terms including ‘thylakoid’, “photosynthesis” and “generation of precursor metabolites and energy” that were over-represented using Fisher’s exact test (FWER < 0.27) among the 15 OGs subject to positive selection (ω > 1) at the 0.05 level (Table 3). These three enriched GO terms involve three OGs, Lhc5 (OG_22685), NDH18 (OG_23367), and PQL2 (OG_23464), respectively (Table S2).

The biochemical pathways of all 550 OGs were investigated using KAAS (KEGG Automatic Annotation Server, http://www.genome.jp/tools/kaas/). According to the results of the KEGG pathway mapping, 139 of the 550 one-to-one OGs could be assigned to 81 pathways. Among these pathways, “Biosynthesis of amino acid”, “Spliceosome”, and “RNA degradation” exhibited the highest number of assigned OGs (Table S4). Among the 15 OGs under positive selection (ω > 1), four were associated with “Photosynthesis”, “Purine metabolism”, and “Pyrimidine metabolism” (Table S4), although they are not statistically significant over-representation (data not shown).

Similar analyses were also applied to the 413 one-to-one OGs in the seven legume species. We found that both models identified the proteins of four OGs as carrying positively selected amino acid sites. They include “alanine-tRNA ligase (OG_9805)”, “ceramide synthase lag1 (OG_9947)”, “acyl-CoA *N*-acyltransferase-like protein (OG_10941)” and “pollen ole e 1 allergen and extensin family protein (OG_10618)” (Table 2 and Table S3).

**Figure 3:**
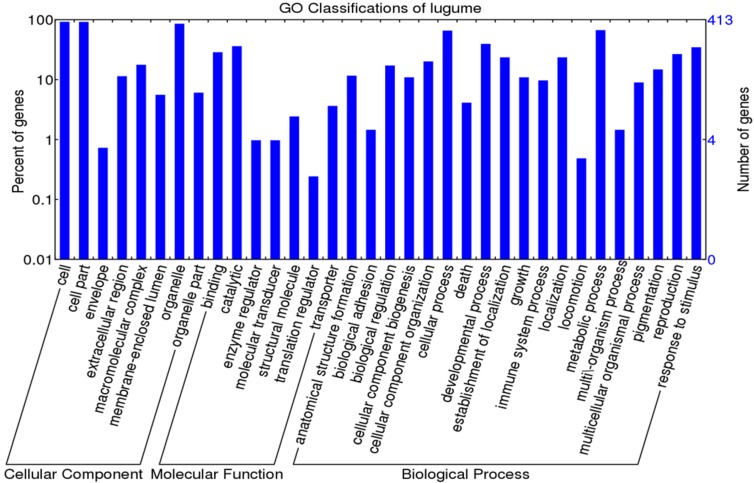
GO classification for 413 OGs of legumes.

Among the 413 one-to-one OGs, a total of 400 OGs were assigned to 36 GO terms (Figure 3 and Table S5). For cellular components, four OGs with positively selected sites were related to “cell part”, “organelle” and “extracellular region”. Regarding molecular functions, these four OGs were related to “ion binding”, “ligase activity” and ‘transferase activity”. As for biological processes, “biosynthetic process”, “primary metabolic process”, “cellular metabolic process”, and “nitrogen compound metabolic process” were mostly enriched (Figure 3 and Table S5). After GO enrichment analysis and a Fisher’s exact test and family wise error rate (FWER) test, the only over-represented term for the four OGs carrying adaptive selected sites was transferring acyl groups (GO:0016746) (Table 3). Two genes including “acyl-CoA *N*-acyltransferase-like protein (OG_10941)” and “ceramide synthase lag1 (OG_9947)” are involved in this term (Table S5).

KEGG Automatic Annotation Server (KAAS) predicted a total of 98 pathways for the 413 OGs with “Biosynthesis of amino acids”, “Carbon metabolism”, and “Spliceosome” occupying the highest number of assigned OGs (Table S6). For the four OGs carrying sites under positive selection in legumes, one metabolite pathway was associated with “aminoacyl-tRNA biosynthesis” (Table S6).

## 3. Discussion

### 3.1. Transcriptome of Lima Bean

With the advent of high-throughput next generation sequencing technologies, studies based on genomic and transcriptomic data for non-model organisms have been increasingly reported [2,3,6,10,25,26,27,28,29]. In this study, we *de novo* assembled lima bean RNA-Seq short reads into a hundred thousand unigenes. RNA-Seq is a cost-effective method for characterizing the transcriptome of non-model species [30], and it provided us an effective tool for exploring complex plant evolutionary biology.

### 3.2. Candidate Adaptive Evolution Genes between Lima Bean and Common Bean

As mentioned previously, the *k*_a_/*k*_s_ ratio is an important index of Darwinian selection. The larger the *k*_a_/*k*_s_ ratio is, the stronger is the positive selection. In our present evolutionary analysis between two *Phaseolus* species, most of the OGs (97.3%; 535/550) had a *k*_a_/*k*_s_ ratio < 1, and this indicates that the majority of OGs were under negative selection (Figure 1). However, we also detected three OGs undergoing adaptive evolution with *k*_a_/*k*_s_ ratios above 2 and 12 OGs with ratios above 1 (Figure 1 and Table S2).

According to the GO enrichment tests, three positively selected OGs, Lhc5, NDH18, and PQL2 were related to three enriched GO terms, “thylakoid”, “photosynthesis” and “generation of precursor metabolites and energy” between the two *Phaseolus* species (Table S2). Lhc5, NDH18 and PQL2 code for parts of subunits of the chloroplast NAD(P)H dehydrogenase (NDH) complex in photosynthesis [31]. Photosynthesis is the most intricate and fundamental physiological process in higher plants, which have developed adaptive mechanisms conferring tolerance to abiotic stresses in the photosynthesis system [32]. PQL has been detected to be under positive selection in homologous pairs of *Arabidopsis* [33]. According to a previous report [34], although lima bean and common bean both originated in tropical and sub-tropical latitudes, they have evolved in different ecological zones; lima bean has evolved at lower altitudes and the common bean at intermediate altitudes. It is likely that the selection pressure of these photosystem-related genes might reflect plant adaptations to different environments.

In addition, a number of OGs under positive selection (ω > 1) between the *Phaseolus* species are involved in resistance to biotic or abiotic stresses. “OG_23307” was identified as under positive selection in the *Phaseolus* species, and it encodes “cold-regulated 413-plasma membrane 2 (COR413-PM)” (Table S2). In plant development of cold tolerance, the COR413-PM protein plays a vital role by stabilizing the plasma membrane [35]. This gene has been detected as under positive selection in two bamboo species [3]. “OG_23031” also exhibited positive selection between the two species. This gene encodes “Seven transmembrane *MLO* family protein” (Table S2). The *MLO* gene family plays an important role in modulating defence responses and cell death during powdery mildew attack [36,37]. In the evolutionary process, many plants fix favourable mutations to enhance their adaptation ability to various biotic and abiotic stresses including pests, pathogens and environmental signals.

### 3.3. Candidate Adaptive Evolution Genes among Seven Legume Species

For the seven legume species, we found that four OGs were identified by both models M1a–M2a and M7–M8, and 13 OGs were determined only by models M7–M8. It is possible that the LRT M1a–M2a model was more robust than M7–M8 [5,38].

The GO enrichment analyses revealed that genes related to transferring acyl groups including acyl-CoA *N*-acyltransferase-like protein (OG_10941) and ceramide synthase lag1 (OG_9947) were probably enriched in legume species (Table 3 and Table S3). The ortholog (OG_10941) encodes an acyl-CoA *N*-acyltransferase-like (Nat) protein. The *A. thaliana* gene (AT1G18335.1) in this OG is Naa40p (Nat 4), which is one of the Nat catalytic subunit isoforms [39,40]. The function of this protein has not been explored in plants. According to studies in humans and yeast [41], Nat 4 is responsible for the acetylation of the N-terminal residues of histones H4 and H2A. Protein N-terminal acetylation is a common covalent modification process of eukaryotic proteins. During evolution, NAT genes could have changed their substrate specificity to adapt to environmental stress. Sabbagh *et al.* [42] have detected positive selection in the NAT gene family during the last 85 million years of primate NAT gene evolution. Another OG (OG_9947) in this enriched term encodes ceramide synthase lag1. Ceramide is the fundamental unit of sphingolipids, which are lipid signalling molecules associated with plant innate immunity [28]. Sphingolipids are associated with plant programmed cell death and plant defence against disease [28]. The adaptive evolution of immune system genes has been detected in many plants [7,43].

Among two other adaptive OGs with sites that had undergone positive selection in seven legume species (Table 2 and Table S3), one OG, Pollen Ole e 1 allergen and extensin family protein (OG_10618), is involved in plant responses to biotic and abiotic stresses. From an evolutionary viewpoint, biotic and abiotic defence genes of plants undergo enhanced positive effects from natural selection under the stress of pathogens, insects and environmental changes. Another OG “OG_9805” encodes alanine-tRNA ligase. The adaptive evolution of alanyl-tRNA synthetase has been reported previously [44].

## 4. Materials and Methods

### 4.1. Sample Collection, cDNA Library Construction, and Illumina Sequencing

The seeds of lima bean (*P. lunatus* cv. Sieva) were provided by Yanhui Lu, Institute of Plant Protection, Chinese Academy of Agricultural Sciences. Lima bean plants (*P. lunatus* cv. Sieva) were kept in a growth chamber (25 ± 2 °C, 50% to 70% R.H., 16L–8D). Two-week old plants’ primary leaves were treated with *Tetranychus cinnabarinus* and Alamethicin, which is a mixture of peptides from the fungus *Trichoderma viride* (*Tetranychus cinnabarinus* control, *Tetranychus cinnabarinus* damage, Alamethicin control, Alamethicin treatment), and the treatments were performed as previously described [45,46]. Samples were collected 24 h later for mRNA extraction. Total RNA was extracted using TRIzol reagent (Invitrogen, Darmstadt, Germany) and treated with RNase-free DNase (Promega, Mannheim, Germany) according to the manufacturer’s protocol. The cDNA library construction and Illumina paired-end sequencing were performed as previously described [29]. The clean Illumina RNA-Seq reads were deposited in the NCBI Sequence Read Archive (SRA, http://www.ncbi.nlm.nih.gov/Traces/sra); accession number SRX894594.

The raw reads were cleaned by removing the adapter sequences and low-quality sequences (containing reads with unknown nucleotides more than 5%, and Phred quality scores less than 20). The sequencing reads for each of the four samples were pooled to perform a single *de novo* transcriptome assembly by Trinity (v. 20140717) [23].

### 4.2. Identification of OGs and Alignment

We downloaded protein-coding and amino acid sequences for *Medicago*, *lotus*, soybean, chickpea, pigeonpea, and common bean from ftp://ftp.jcvi.org/pub/data/m_truncatula/Mt4.0/, ftp://ftp.Kazusa.or.jp/pub/lotus/lotus_r2.5/, ftp://ftp.jgi-psf.org/pub/JGI_data/phytozome/v10.0/Gmax/, http://www.ncbi.nlm.nih.gov/genome/?term=PRJNA175619, http://www.ncbi.nlm.nih.gov/bioproject/?term=PRJNA72815, and http://phytozome.jgi.doe.gov/pz/portal.html#; !info? alias=Org_Pvulgaris V1.0, respectively. Data for the out-group species (*Arabidopsis thaliana*) were acquired from ftp://ftp.arabidopsis.org/home/tair/Genes/TAIR10_genome_release/.

The OGs of the eight species were determined following the method of Zhao *et al.* [3]. Briefly, we assigned one-to-one core-orthologs from all “primer taxa” that consist of eight whole-proteome species including *Medicago*, *lotus*, soybean, chickpea, Pigeonpea, common bean and *A. thaliana* by OrthoMCL5 [47]. Then, one-to-one core-orthologs were put into HaMStRv13.2.3 [48] to search for the corresponding OG in the lima bean transcriptome data.

To filter possible paralogs, we searched pairs of sequences against protein sequences in GenBank, and then only those mapping to the identical protein with an *E*-value cutoff of 1 × 10^−15^ were assigned as OGs according to methods and criteria adopted in previous studies [10,25,27,49].

For each OG, The BLAST-Like Alignment Tool (BLAT) [50] was used to generate a pair-wise codon-based alignment, and then it was checked with MEGA6 [51] for further Phylogenetic Analysis by Maximum Likelihood (PAML) analysis [52]. We excluded alignments that were shorter than 90 bp from the data set to avoid introducing biases into the analysis due to the low number of substitutions in some genes.

### 4.3. Detection of Selection Analysis

We used the codeml programme under the F3 × 4 model of the PAML 4.7 package [52] to estimate positive selection OGs between the two *Phaseolus* species (lima bean and common bean) and across the seven legume species. To estimate selective pressure on the 550 OGs between lima bean and common bean, we performed pairwise maximum likelihood analysis with runmode to −2 and NSsites to 0 in PAML 4.7. According to previous studies [10,24], ω > 1, ω = 1 and ω < 1 suggest positive (adaptive) selection, neutral evolution and negative (purifying) selection, respectively.

To further test amino acid sites that had undergone positive selection, we performed site models using runmode 0 and four models, M1a (nearly neutral), M2a (positive selection), M7 (β) and M8 (β + ω) on the 413 OGs from seven legume species. LRT [38,53] were carried out to identify positive selection sites between M1_a_ and M2_a_ (2 d.f.) and between M7 and M8 (2 d.f.), which were then compared against the χ^2^ distribution. We next employed FDR to correct multiple testing by the Benjamini and Hochberg method [54]. When the LRT and FDR were significant (<0.05), BEB [55] was then used to examine amino acid sites under positive selection (BEB value > 95%). The tree of every OG used by the CodeML programme was built by maximum likelihood using RAxML-7.2.8-ALPHA.

### 4.4. Function Annotation

The *A. thaliana* ortholog of each OG was used to perform Gene Ontology (GO) and KEGG pathway annotation for all the OGs. Functional annotation of the unigenes were achieved by searching against the nr and Swiss-Prot databases using BLASTx with *E*-value < 1 × 10^−5^. The blast output was imported into Blast2GO for GO annotation [56,57]. WEGO software was then used [58] to perform GO functional classification for all the unigenes at the macro level. By setting the original OGs as the reference dataset and the positively selected genes as the test dataset, GO term enrichment between the reference and test datasets was determined using FWER. The analysis was performed by the GOSSIP software package [59]. KEGG pathway mapping was conducted on the Web of KAAS (http://www.genome.jp/tools/Kaas/).

## 5. Conclusions

In the current study, we observed 15 and four OGs with signatures of positive selection among 550 and 413 OGs in two *Phaseolus* species and seven legume species, respectively. Functional annotation of these positively selected genes revealed that they are mostly involved in thylakoids, photosynthesis and metabolism. These genes may be related to the divergence of the *Phaseolus* and legume species. The genes identified in this study as being under positive selection are particularly good candidates for subsequent functional and evolutionary studies. Several genes are interesting candidates to investigate for plant adaptation to biotic and abiotic stresses, and they could serve as gene pool for novel biotic and abiotic resistance that may enhance legume breeding programmes.

## Supplementary Materials

Supplementary materials can be found at http://www.mdpi.com/1422-0067/16/07/15172/s1.
